# Concentrations of Serum Brain Injury Biomarkers Following SARS-CoV-2 Infection in Individuals with and without Long-COVID—Results from the Prospective Population-Based COVI-GAPP Study

**DOI:** 10.3390/diagnostics13132167

**Published:** 2023-06-26

**Authors:** Julia Telser, Kirsten Grossmann, Ornella C. Weideli, Dorothea Hillmann, Stefanie Aeschbacher, Niklas Wohlwend, Laura Velez, Jens Kuhle, Aleksandra Maleska, Pascal Benkert, Corina Risch, David Conen, Martin Risch, Lorenz Risch

**Affiliations:** 1 Dr. Risch Medical Laboratory, 9490 Vaduz, Liechtenstein; julia.telser@risch.ch (J.T.); kirsten.grossmann@blutdruck.li (K.G.); ornella.weideli@blutdruck.li (O.C.W.); stefanie.aeschbacher@usb.ch (S.A.); niklas.wohlwend@risch.ch (N.W.); laura.velez@risch.ch (L.V.); corina.risch@risch.ch (C.R.); martin.risch@risch.ch (M.R.); 2 Dr. Risch Medical Laboratory, 9470 Buchs, Switzerland; dorothea.hillmann@risch.ch; 3 Faculty of Medical Sciences, Private University in the Principality of Liechtenstein (UFL), 9495 Triesen, Liechtenstein; 4 Soneva Fushi, Boduthakurufaanu Magu, Male 20077, Maldives; 5 Department of Cardiology, University Hospital of Basel, 4031 Basel, Switzerland; 6 Faculty of Medicine, University of Bern, 3012 Bern, Switzerland; 7 University of St. Gallen, 9000 St. Gallen, Switzerland; 8 Neurologic Clinic and Policlinic, MS Center and Research Center of Clinical Neuroimmunology and Neuroscience Basel (RC2NB), University Hospital Basel, University of Basel, 4031 Basel, Switzerland; jens.kuhle@usb.ch (J.K.); aleksandra.maleska@usb.ch (A.M.); pascal.benkert@usb.ch (P.B.); 9 Population Health Research Institute, McMaster University, Hamilton, ON L8L 2X2, Canada; david.conen@phri.ca; 10 Division of Laboratory Medicine, Kantonsspital Graubünden, 7000 Chur, Switzerland

**Keywords:** COVID-19, SARS-CoV-2, serum biomarker, glial fibrillary acidic protein, neurofilament light chain, GFAP, NfL

## Abstract

It is unknown whether neurological symptoms are associated with brain injury after SARS-CoV-2 infections and whether brain injury and related symptoms also emerge in Long-COVID patients. Biomarkers such as serum neurofilament light chain (sNfL) and glial fibrillary acidic protein (sGFAP) can be used to elucidate neuro-axonal and astroglial injuries. We investigated whether these biomarkers are associated with COVID-19 infection status, associated symptoms and Long-COVID. From 146 individuals of the general population with a post-acute, mild-to-moderate SARS-CoV-2 infection, sNfL and sGFAP were measured before, during and after (five and ten months) the infection. Individual symptoms and Long-COVID status were assessed using questionnaires. Neurological associated symptoms were described for individuals after a mild and moderate COVID-19 infection; however, sNfL (*p* = 0.74) and sGFAP (*p* = 0.24) did not change and were not associated with headache (*p* = 0.51), fatigue (*p* = 0.93), anosmia (*p* = 0.77) or ageusia (*p* = 0.47). In Long-COVID patients, sGFAP (*p* = 0.038), but not sNfL (*p* = 0.58), significantly increased but was not associated with neurological associated symptoms. Long-COVID status, but not post-acute SARS-CoV-2 infections, may be associated with astroglial injury/activation, even if neurological associated symptoms were not correlated.

## 1. Introduction

During the severe acute respiratory syndrome coronavirus 2 (SARS-CoV-2) pandemic, mild-to-severe neurological complications were reported [1,2]. Such neurological symptoms are often reported during the acute phase of the infection [3], but there is increasing evidence that they may persist for months, unrelated to the infection’s initial severity [4]. This lasting symptom burden in individuals with a history of a SARS-CoV-2 infection, usually three months from the onset, with symptoms that last for at least two months and cannot be explained by an alternative diagnosis, has led the World Health Organization (WHO) to establish a post-COVID-19 syndrome, also known as Long-COVID [5].

Ultrasensitive Single Molecular Array (Simoa) Assays allow the detection of diverse cerebrovascular injury in blood samples of acute and post-acute COVID-19 patients with high accuracy [6]. One of those brain injury biomarkers is the glial fibrillary acidic protein (GFAP), which is the major protein component of the glial intermediate filaments in astrocytes [7] and regulates the function of these cells [8]. In response to central nervous system (CNS) injuries, astrocytes proliferate and increase in size and there is a substantial increase of glial filaments and GFAP content [7]. As astrocytes are cells that make up to blood–brain barrier, damage to the blood–brain barrier will cause GFAP levels to increase and enter the bloodstream [9]. Therefore, astrocytic damage or activation can be indicated by increased GFAP levels in the blood [8].

Other biomarkers include neurofilaments (Nfs), exclusively expressed in neurons of the central and peripheral nervous system and conferring structural stability to neurons [10,11]. In the CNS, Nfs are made of neurofilament light chain (NfL), neurofilament middle chain (NfM), neurofilament heavy chain (NfH) and α-internexin (α-int) [12]. Under normal conditions, low levels of NfL are constantly released from axons. However, in response to CNS axonal damage because of inflammatory, neurodegenerative, traumatic or vascular injury, the release of NfL increases [12]. The released NfL reaches the interstitial fluid, which communicates freely with the CSF [12]. Since NfL has the lowest molecular weight, it can diffuse from parenchyma to cerebrospinal fluid (CSF) and blood [6], making it a useful biomarker for neuro-axonal injury [6].

Recently, elevated levels of GFAP and NfL were found during the acute phase in blood samples of patients with severe (hospitalization with intensive care unit (ICU)) SARS-CoV-2 infections [13,14,15,16,17]. However, despite the large number of patients with mild-to-moderate SARS-CoV-2 symptoms [18], only a few studies have investigated blood markers of brain injury in these groups during or even after resolution of the acute phase of the infection. One of these studies showed increased serum NfL (sNfL) and serum GFAP (sGFAP) levels in non-hospitalized adolescents [19], while another study reported increased sNfL levels in adult health-care workers with mild-to-moderate symptoms [20]. However, up to date it is still poorly understood whether the increase in the investigated biomarkers is associated with neurological symptoms in these groups. Therefore, to detect possible associations of sGFAP, sNfL and neurological associated symptoms and to assess whether biomarkers longitudinally change over time, we performed a follow-up study of patients after mild-to-moderate SARS-CoV-2 infections. Additionally, we investigated whether sGFAP and sNfL change in Long-COVID patients after a mild-to-moderate infection, and whether these biomarkers are associated with Long-COVID symptoms.

## 2. Materials and Methods

### 2.1. Study Design and Participants

All participants from the population-based prospective cohort study (genetic and phenotypic determinants of blood pressure and other cardiovascular risk factors *n* = 2170) [21] were invited to participate in the sub-study COVI-GAPP. The COVI-GAPP study was initiated to investigate the use of a sensor bracelet (Ava-bracelet) to identify pre-symptomatic SARS-CoV-2 infections and detect infection-related physiological changes [22]. Over the study period from 2020 to 2022, COVI-GAPP participants (*n* = 1144) were invited four times for blood collections at the study center in Vaduz, Lichtenstein.

**Figure 1:**
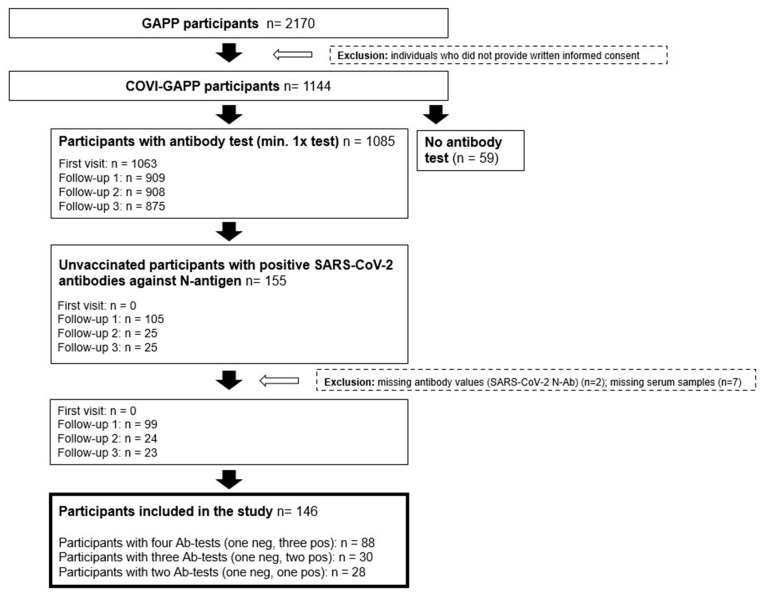
Study flow chart. From 2170 GAPP participants, 1144 participants were enrolled in the COVI-GAPP study. A total of 155 participants were unvaccinated and had a negative (control) and a minimum of one positive antibody-value against the SARS-CoV-2 nucleocapsid (N) antigen. After exclusion of nine participants due to missing data or serum samples, a total of 146 participants were included in the study. From those, 88 participants had four tests (one negative, three positive), 30 participants had three tests (one negative, two positive) and 28 participants had two tests (one negative, one positive).

In the current study, a post-acute SARS-CoV-2 infection was diagnosed with positive antibody results against the nucleocapsid (N) antigen of the SARS-CoV-2 virus. Only SARS-CoV-2-unvaccinated participants at the time of infection with a negative (control) and a minimum of one positive antibody value against the SARS-CoV-2 N-antigen indicating seroconversion due to infection were included. From these, two participants were excluded due to missing baseline antibody values against the SARS-CoV-2 nucleocapsid (N) antigen, and seven participants were excluded due to insufficient sample volume, resulting in 146 participants with confirmed infection (Figure 1). From those, 88 participants had four tests (one negative, three positive), 30 participants had three tests (one negative, two positive) and 28 participants had two tests (one negative, one positive; Figure 1). Three participants could not be reached for clinical follow-up information, leading to 143 symptom queries for the respective analyses.

From the 146 included participants, 133 participants had a mild infection, while 13 participants had a moderate infection. A mild infection is here defined as an infection that did not require hospitalization, while a moderate infection required hospitalization, but no intensive care unit (ICU).

Thirty-nine participants with a continuation or development of new symptoms three months after the initial SARS-CoV-2 infection with these symptoms lasting for at least two months were considered as having Long COVID, as defined by the WHO. From those participants, biomarker analysis (sNfL/sGFAP) was performed with the serum sample taken at the same time when participants first reported Long-COVID symptoms.

Informed written consent was obtained from each participant, and the local ethics committee (KEK, Zürich, Switzerland) approved the study protocol (BASEC 2020-00786).

### 2.2. Blood Sample Collection

Study-related blood collection only took place once participants were free of symptoms of active COVID-19 infection. At each visit, a venous blood sample was obtained from participants by trained study nurses in a standardized manner [21]. Serum samples were kept at room temperature (RT) before SARS-CoV-2 antibody (SARS-CoV-2-N-Ab and SARS-CoV-2-S1-Ab) analysis, which took place within 24 h after blood collection. One aliquot was subsequently stored at −25 °C before it entered a biobank for long-term storage at −80 °C.

### 2.3. SARS-CoV-2 Antibody Measurement

SARS-CoV-2 antibody tests were assessed by Dr. Risch Ostschweiz AG, Buchs SG, Switzerland, an ISO-17025-accredited medical laboratory. Antibody levels were determined by electrochemiluminescence immunoassay (ECLIA) using the Elecsys^®^ Anti-SARS-CoV-2 immunoassays (Roche Diagnostics, Rotkreuz, Switzerland) measured on a COBAS 6000. The Elecsys^®^ Anti-SARS-CoV-2 S assay uses a recombinant protein representing the receptor-binding domain (RBD) of the spike (S) antigen or the nucleocapsid (N) antigen in a double-antigen sandwich assay format, which favors detection of high-affinity pan-immunoglobulins directed against these SARS-CoV-2 antigens.

### 2.4. Serum GFAP and Serum NFL Measurements

GFAP and NfL measures were performed at a median of two months (60 days; IQR: 32.0 to 77.5) after an acute infection. For GFAP and NfL analyses, the samples from the biobank were thawed, vortexed and aliquoted. Then, they were frozen and shipped on dry ice to the University Hospital Basel, Switzerland, for serum glial fibrillary acidic protein (sGFAP) and serum neurofilament light chain (sNfL) analysis [23]. sGFAP and sNfL measurements were performed with the commercially available Simoa Human Neurology 2-Plex B assay (N2PB, Item 103520) from Quanterix (Quanterix, Billerica, MA, USA) on the HD-X Simoa platform. Samples were analyzed in duplicate determination according to the manufacturer’s instructions.

All samples were analyzed by technicians blinded to the SARS-CoV-2 antibody values and health status of the participants. All sample concentrations were higher than the concentration of the lowest calibrator and lower than the concentrations of the highest calibrator. For GFAP, the mean inter-assay coefficient of variation (CV) of internal QCs was 9.7% (low), 10.6% (medium) and 9.0% (high); and for NfL, was 4.3% (low), 1.5% (medium) and 9.9% (high). For GFAP, the mean intra-assay CV from duplicate determination was 2.8%, and for NfL, the mean intra-assay CV was 5%. Intra-assay coefficients of variation were below 15% for all analyses.

### 2.5. Questionnaires

At the follow-up blood collections (five months and ten months post-infection), participants were asked to complete a written questionnaire, providing information about vaccination and infection status, and the duration of persistent symptoms (Long-COVID symptoms).

**Figure 2:**
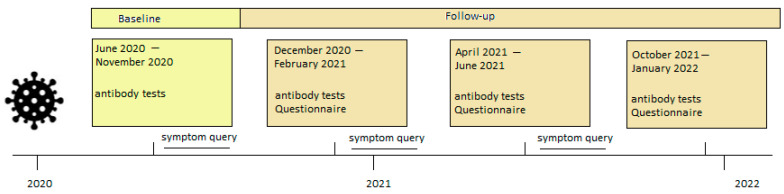
Timeline of data collection. Each participant was invited for four antibody tests: An initial blood test (begun June 2020, light yellow) and three follow-up blood tests (dark yellow). In each follow-up blood collection, a questionnaire was completed by the participants.

If participants had any symptoms during the study period, they were encouraged to visit the Liechtenstein National Testing Facility for reverse transcription-polymerase chain reaction (RT-PCR) testing, which was performed with either the COBAS 6800 platform (Roche Diagnostics, Rotkreuz, Switzerland) or the TaqPath assay on a QuantStudio 5 platform (Thermo Fisher Scientific, Allschwil, Switzerland) [24,25,26]. Positively tested participants (PCR and antibody tests, or only antibody tests) were subsequently contacted by the study team and asked to report their symptoms (fever, fever degree, chills, cough, sniff, dyspnea, anosmia, ageusia, pressure in the chest, sore throat, muscle pain, headache, fatigue, general feeling of illness, diarrhea, sickness, vomiting) and hospitalization status by a standardized questionnaire commissioned by telephone interview (Figure 2).

### 2.6. Statistical Analysis

We conducted the Mann–Whitney U test and chi-square test for demographics, stratified by infection severity (mild infection without hospitalization or moderate infection with hospitalization but no ICU). Distributions were assessed by visual inspection and outliers were detected using the Grubbs-right- and Grubbs-left-sided (alpha-level 0.05) test. The Wilcoxon test was used to assess biomarker (sNfL and sGFAP) differences between the SARS-CoV-2-N-Ab-negative and SARS-Cov-2-N-Ab-positive group. To assess differences between the Long-COVID groups (WHO definition) vs. no Long-COVID group, the Mann–Whitney U test was used. To compare biomarker differences over time (follow-up of five months and ten months), we conducted the Friedman repeated measures test. To assess whether the reported symptoms are associated with brain injury biomarkers after a mild-to-moderate SARS-CoV-2 infection and with Long-COVID status, the Mann–Whitney U test was used. The Wilcoxon test was used to assess groupwise biomarker differences in participants with neurological associated symptoms before and after a SARS-CoV-2 infection. In addition, to assess whether sNfL and sGFAP are independently associated with COVID-19 status, a repeated measures ANOVA or a multivariable adjusted linear regression analysis was used, with sNfL and sGFAP as dependent log-transformed variables, and age, sex and COVID-19 status as independent covariates. Outlier Analysis was conducted using MedCalc Version 20.027 and RStudio 2021.09.0 und R version 4.1.3.

## 3. Results

### 3.1. Demographics

Table 1 contains overall participant characteristics and clinical variables, overall and stratified by infection severity. Study participants were mainly infected by wild type, B.1.258 or B.1.1.7 variants, and B1.617.2. SARS-CoV-2 infections with Omicron were not observed within this period. The entire cohort (mildly and moderately infected participants combined) consisted of 146 unvaccinated participants at the time of infection, among which, 57% were vaccinated (87.5% one shot and 12.5% two shots, no differences in sNfL or sGFAP levels in vaccinated vs unvaccinated participants, data not shown) during the follow-up time. Reinfection was reported in 1.4% of cases in the entire cohort. The median age of all participants was 43.2 years (SD = 5.6), and 88 (60%) participants were female. From the total of 146 individuals, 91% (133 participants) had a mild SARS-CoV-2 infection (without hospitalization) and 9% (13 participants) had a moderate SARS-CoV-2 infection (with hospitalization, but no ICU). There was no statistically significant difference between groups in terms of age (*p* = 0.13) and sex (*p* = 0.62). The duration of the acute SARS-CoV-2 infection was significantly longer in participants with a moderate infection compared to those with a mild infection (*p* < 0.0001). Participants with a moderate infection were suffering more frequently from Long COVID (*p* = 0.01). The most reported symptoms for the entire cohort were headache (66%) and fatigue (57%). Participants with a mild infection reported fewer symptoms (1–5 symptoms) than participants with a moderate infection (11–15 symptoms) and most queried symptoms were reported more frequently in participants with a moderate infection. We found no statistical difference in the occurrence of neurological associated symptoms such as headache, fatigue, anosmia and ageusia in patients with mild or moderate SARS-CoV-2 infections (Table 1).

sNfL was highly associated with participants’ age but not with participants’ sex (Table 2). For sGFAP, no association was found with either age or sex (Table 2). Since brain injury biomarkers are dependent on age, in all further analysis, the association between COVID-19 status and brain injury biomarkers was determined using a repeated measures ANOVA or multivariable adjusted linear regression analysis with sNfL or SGFAP as dependent log-transformed variables, and age, sex and COVID-19 status as independent variables. Moreover, no correlation was found between antibody concentrations (SARS-CoV-2-N-Ab) with neither NfL nor GFAP biomarker levels (data not shown).

### 3.2. Biomarkers of Brain Injury after a Mild-to-Moderate SARS-CoV-2 Infection 

**Figure 3:**
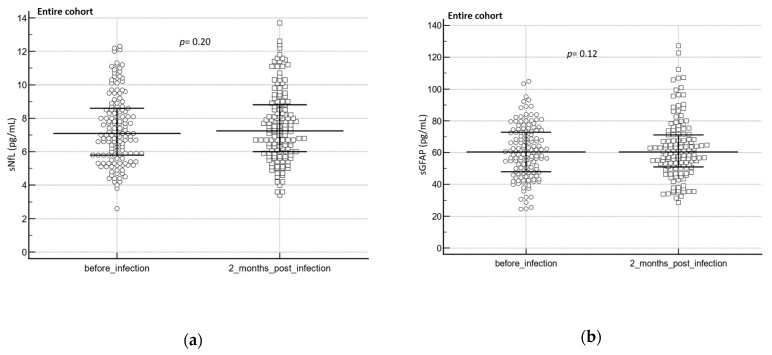
Brain injury biomarkers (sNfL/sGFAP) before and after a SARS-CoV-2 infection and at different clinical groups. (**a**) sNfL before and after infection (*n* = 142), (**b**) sGFAP before and after infection (*n* = 140). The Wilcoxon test was used to assess group differences before and after an infection. Complete cases are represented in the dot plot. The central horizontal line represents the median with the interquartile range (IQR).

In the entire cohort, neither sNfL (*p* = 0.20) nor sGFAP (*p* = 0.12) levels significantly changed after an infection (two months post-infection, median: 60 days, IQR: 32.0 to 77.5; Figure 3a,b).

**Figure 4:**
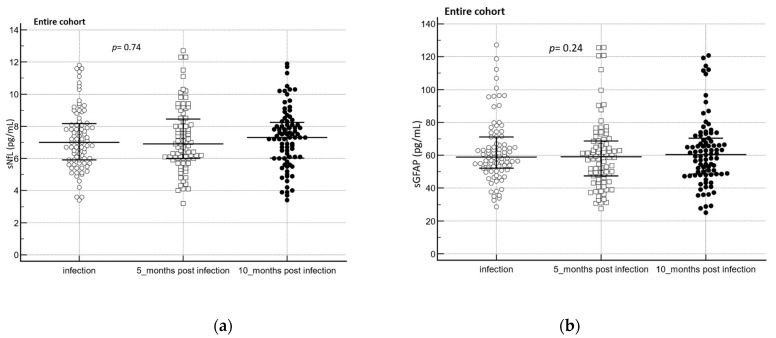
(**a**) sNfL (*n* = 83) and (**b**) sGFAP (*n* = 84) levels over time in the entire cohort. To assess biomarker difference between the groups, the Friedmann test was used. Complete cases are represented in the dot plot. The central horizontal line represents the median with the interquartile range (IQR).

Over a follow-up period of ten months post-infection, sNfL (*p* = 0.74) and sGFAP (*p* = 0.24) levels did not change significantly (Figure 4a,b).

In participants from the entire cohort suffering from neurological associated symptoms, sNfL and sGFAP levels did not change after an infection (Table 3).

Although reported frequently during SARS-CoV-2 infection, the occurrence of neurological associated symptoms such as headache, fatigue, ageusia and anosmia was not associated with sNfL or sGFAP levels in the entire cohort (Table 4).

### 3.3. Biomarkers of Brain Injury in Participants with Long COVID

**Figure 5:**
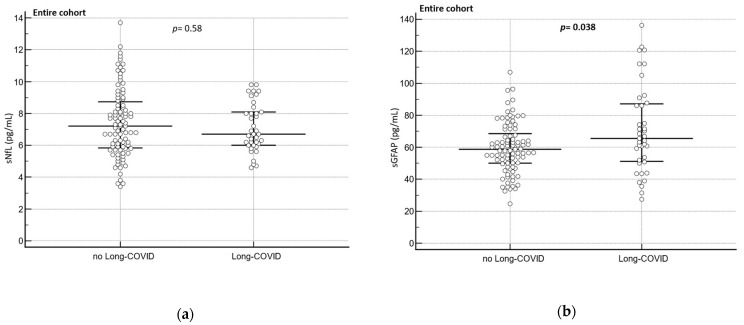
Brain injury biomarkers (sNfL/sGFAP) in patients with and without Long COVID at different clinical groups. (**a**) sNfL in patients without vs. with Long COVID (*n* = 91 vs. 38), (**b**) GFAP in patients without vs. with Long COVID (*n* = 89 vs. 39). The Mann–Whitney U Test was used to assess the biomarker difference between the groups. Complete cases are represented in the dot plot. The central horizontal line represents the median with the interquartile range (IQR).

No difference in sNfL levels were found between participants with Long COVID (*n* = 38) and participants without Long COVID (*n* = 91; *p* = 0.58)) in the entire cohort (Figure 5a). Conversely, participants from the entire cohort with Long COVID (*n* = 39) had significantly higher sGFAP levels compared to those participants without Long COVID (*n* = 89; *p* = 0.038, Figure 5b).

In participants with Long COVID suffering from neurological associated symptoms such as headache, fatigue anosmia and ageusia, sNfL and sGFAP levels did not change after an infection (Table 5).

Neurological associated symptoms such as headache, fatigue, ageusia and anosmia were not associated with sNfL or sGFAP in Long-COVID patients (Table 6).

## 4. Discussion

In the present study, we show that participants with a moderate SARS-CoV-2 infection have a longer-lasting acute infection phase accompanied by several symptoms, and are affected more frequently by Long COVID than mildly affected participants. Both sub-groups reported neurological associated symptoms, but the frequency did not vary between the groups.

These reported neurological associated symptoms were not associated with serum markers of brain injury (sNfL/sGFAP), and in line with this, no association was found between the infection status and brain injury biomarkers, at least not at the investigated time points.

Together, these results suggest that COVID-19 infection and potentially associated neurological symptoms may not leave biochemical traces of neuro-axonal or astroglial damage after resolution in disease, at least not in peripheral blood samples. However, it cannot be ruled out that the biomarkers may have been abnormal when measured in the more sensitive CSF samples.

Our results from non-ICU individuals after a mild-to-moderate infection suffering from Long COVID indicate that sGFAP levels were related to Long COVID. Our study therefore reported for the first time that Long-COVID status may be associated with brain injury. Interestingly, this seems to be a unique feature of sGFAP, since no relation was found for sNfL in our cohort. This may suggest that the origin of some symptoms related to Long COVID differ from those symptoms coinciding with the acute or post-acute phase of the COVID-19 disease. Moreover, persisting symptoms do not necessarily mean persistence of a pathological brain damaging process, which may explain the missing correlation with sNfL.

The lack of association of serum biomarkers of brain injury and infection status differs from previous studies. For example, Ameres et al. [20] found NfL to be elevated in adult health-care workers with a mild-to-moderate COVID-19 infection, and Havdal et al. [19] found an increase in NfL and GFAP in non-hospitalized adolescents. The differing results may be explained by the larger sample size in the study from Havdal et al. [19], resulting in a more powered study that is more likely to detect significant correlations. Moreover, compared to our study, Havdal et al. [19] and Ameres et al. [20] used a different study design (with a control group) and investigated biomarkers in the acute phase of the SARS-CoV-2 infection (Havdal et al. [19] not more than 28 days since the first day of symptoms or positive PCR test, and Ameres et al. [20] at 23 days after the onset of the disease), whereas our study investigated brain injury biomarkers in individuals in the post-acute stage of the infection (60 days after the infection). Prior studies investigating blood biomarkers of brain injury in other conditions than COVID-19 revealed that GFAP and NfL levels can remain elevated months to years after the injury, with fluctuating elevations over time [27]. Therefore, it cannot be ruled out that biomarkers may have changed when measured at other time points of the infection. 

In addition, Havdal et al. [19] included participants with an age between 12 and 25 years, whereas the median age of our participants was 43.2 years. Since it is known that the severity of COVID-19 differs with age [28], it cannot be excluded that this factor may influence brain injury biomarker levels. 

Lastly, it cannot be excluded that there were some pre-analytical differences in sample handling between the studies that might affect the results. Even if for most pre-analytical variables, serum NfL and GFAP levels remain unaffected, NfL values can be influences by delayed centrifugation [29].

The ultrasensitive measurements of brain injury biomarkers in blood samples allow one to monitor minor changes in protein levels, enabling one to assess neurological damage with a minimal invasive procedure [10]. This may be helpful in identifying various pathophysiological changes associated with COVID-19 infections, which would be critical to understand the course of the disease. Especially in Long COVID, this may be helpful in patient management in clinical practice. 

However, before the implementation of blood–brain injury biomarkers in routine clinical laboratories, it is essential to validate the different biomarkers in COVID-19 patients and in patients with Long COVID in larger studies to make them suitable for clinical settings. In addition, the investigation of other CNS-derived analytes might be important to enhance the accuracy of non-invasive monitoring of CNS disorders in COVID-19.

### Strengths and Limitations

Strengths of our study include a well-defined group of unvaccinated individuals from the general population with a mild-to-moderate course of COVID-19 infection. Moreover, our study included longitudinal data with a follow-up period of 10 months post-infection and participants with a mild-to-moderate COVID-19 infection suffering from Long COVID (WHO definition).

A weakness of the study Is the small sample size of participants with a moderate infection not allowing subgroup analysis according to disease severity. In addition, symptoms are self-reported and therefore, vulnerable to recall bias. Furthermore, detailed clinical data during hospitalization, such as oxygen status, were not available. In addition, we do not have any data of the acute phase of the infection, allowing an investigation of the markers only in post-acute settings. However, we do not believe that these limitations invalidate our findings.

## 5. Conclusions

Post-acute, mild-to-moderate COVID-19 cases from the general population showed no association with serum brain injury biomarkers, and neurological associated symptoms may not be a result of neuro-axonal or astroglial damage in those individuals. Individuals with a mild-to-moderate infection suffering from Long COVID showed an increase in serum biomarkers of astroglial injury, but not neuro-axonal damage. Therefore, our study reported for the first time that Long-COVID status, but not a post-acute SARS-CoV-2 infection, may be associated with brain injury. To draw further conclusions and strengthen the evidence, additional studies in individuals with mild-to-moderate COVID-19 infection, with and without Long COVID and sNfL/sGFAP, are required.

## Figures and Tables

**Table 1:** Demographics, serum biomarkers and symptoms. Data are shown as mean (SD) or *n* (%). Statistically significant difference was set at *p* < 0.05 (bold type). * In the category “Duration acute SARS-CoV-2 infection”, four data points are missing. Total: *n* = 142; mild infection: *n* = 129; moderate infection: *n* = 13. The Mann–Whitney U test and chi-square test were used to assess group differences.

Variables	Entire Cohort *n* = 146	Mild Infection (No Hospitalization) *n* = 133	Moderate Infection (Hospitalization) *n* = 13	*p*-Value
Age, years (SD)	43.2 (5.6)	43.4 (5.4)	41.5 (6.1)	*p* = 0.13
Sex, female (%)	88 (60%)	81 (61%)	7 (54%)	*p* = 0.62
Duration acute SARS-CoV-2 infection, days (SD) *	9.6 (6.73)	8.3 (5.04)	22 (8)	***p* < 0.0001**
Long COVID-19 (%)	39 (26%)	30 (23%)	9 (69%)	***p* = 0.01**
Symptoms quantity 1–5	77 (54%)	74 (57%)	3 (23%)	***p* = 0.02**
Symptoms quantity 6–10	56 (39%)	52 (40%)	4 (31%)	*p* = 0.51
Symptoms quantity 11–15	10 (7%)	4 (3%)	6 (46%)	***p* < 0.0001**
Headache (%)	97 (66%)	87 (67%)	10 (78%)	*p* = 0.46
Fatigue (%)	83 (57%)	74 (57%)	9 (69%)	*p* = 0.39
Arthralgia (%)	71 (49%)	61 (47%)	10 (78%)	***p* = 0.04**
Anosmia (%)	66 (45%)	58 (45%)	8 (62%)	*p* = 0.24
Ageusia (%)	55 (38%)	48 (37%)	7 (54%)	*p* = 0.23
Fever (%)	51 (35%)	41 (31.5%)	10 (77%)	***p* = 0.001**
Chills (%)	36 (25%)	28 (22%)	8 (62%)	***p* = 0.001**
Cough (%)	65 (45%)	56 (43%)	9 (70%)	*p* = 0.07
Rhinitis (%)	58 (40%)	52 (40%)	6 (46%)	*p* = 0.67
Dyspnea (%)	23 (16%)	18 (14%)	5 (38%)	***p* = 0.02**
Thoracic tightness (%)	22 (15%)	14 (11%)	8 (62%)	***p* < 0.0001**
Sore throat (%)	40 (27%)	34 (26%)	6 (46%)	*p* = 0.13
General illness (%)	65 (45%)	55 (42%)	10 (77%)	***p* = 0.02**
Diarrhea (%)	28 (19%)	22 (17%)	6 (46%)	***p* = 0.01**
Sickness (%)	22 (15%)	16 (12%)	6 (46%)	***p* = 0.001**
Vomiting (%)	8 (5%)	3 (2%)	5 (38%)	***p* < 0.0001**

**Table 2:** Association of sNfL and sGFAP with age and sex. Multiple regression analysis of sNfL and sGFAP as dependent variables and age and sex as independent variables. Statistically significant difference was set at *p* < 0.05 (bold type).

	sNfL		sGFAP	
Variables	Coefficient (Sth. Error)	*p*-Value	Coefficient (Sth. Error)	*p*-Value
Age	b1 = 0.11 (0.03)	***p* = 0.002**	b1 = 0.54 (0.35)	*p* = 0.12
Sex	b1 = 0.17 (0.43)	*p* = 0.78	b1 = 5.27 (4.26)	*p* = 0.22

**Table 3:** sNfL and sGFAP in the entire cohort with neurological associated symptoms such as headache (sNfL *n* = 93, sGFAP *n* = 91), fatigue (sNfL *n* = 78, sGFAP *n* = 75), anosmia (sNfL *n* = 64, sGFAP *n* = 60) and ageusia (sNfL *n* = 54, sGFAP *n* = 50) prior to a SARS-CoV2 infection and during the infection period. The Wilcoxon test was used to assess group differences.

	sNfL before Infection vs. after Infection			sGFAP before Infection vs. after Infection		
Symptoms	Median	IQR	*p*-Value	Median	IQR	*p*-Value
headache	7.1 vs. 7.2	5.7 to 8.7 vs. 5.9 to 8.8	*p* = 0.25	60.5 vs. 60.4	47.6 to 72.2 vs. 51.3 to 68.1	*p* = 0.51
fatigue	7.2 vs. 7.2	5.9 to 9.2 vs. 5.8 to 8.5	*p* = 0.84	61.4 vs. 59.4	47.6 to 72.2 vs. 51.1 to 67.2	*p* = 0.93
anosmia	12.3 vs. 12.6	5.8 to 9.0 vs. 5.7 to 8.8	*p* = 0.88	60.7 vs. 58.7	49.4 to 72.7 vs. 51.3 to 67.4	*p* = 0.77
ageusia	7.2 vs. 7.2	5.9 to 8.9 vs. 5.6 to 8.1	*p* = 0.73	61.1 vs. 56.9	47.9 to 75.5 vs. 46.9 to 66.3	*p* = 0.47

**Table 4:** sNfL and sGFAP (median and IQR) in participants with vs. without headache (sNfL *n* = 95 vs. 46, sGFAP *n* = 90 vs. 45), fatigue (sNfL *n* = 81 vs. 60, sGFAP *n* = 78 vs. 60), anosmia (sNfL *n* = 65 vs. 76, sGFAP *n* = 61 vs. 74) and ageusia (sNfL *n* = 54 vs. 87, sGFAP *n* = 51 vs. 85). The Mann–Whitney U Test was used to assess biomarker difference between the groups.

	sNfL			sGFAP		
Symptoms	Median	IQR	*p*-Value	Median	IQR	*p*-Value
headache vs. no headache	7.2 vs. 7.3	5.9 to 8.8 vs. 6.0 to 9.0	*p* = 0.89	60.4 vs. 58.4	50.9 to 68.3 vs. 50.0 to 72.2	*p* = 0.99
fatigue vs. no fatigue	7.2 vs. 7.2	5.8 to 8.5 vs. 6.2 to 9.0	*p* = 0.69	59.7 vs. 60.2	50.3 to 67.3 vs. 50.1 to 75.9	*p* = 0.38
anosmia vs. no anosmia	7.4 vs. 7.2	5.7 to 8.8 vs. 6.5 to 8.9	*p* = 0.99	58.5 vs. 60.2	49.4 to 67.3 vs. 50.1 to 71.5	*p* = 0.41
ageusia vs. no ageusia	7.2 vs. 7.2	5.6 to 8.1 vs. 6.1 to 9.1	*p* = 0.25	56.2 vs. 61.0	44.7 to 65.9 vs. 50.7 to 71.5	*p* = 0.09

**Table 5:** sNfL and sGFAP in Long-COVID patients with neurological associated symptoms such as headache (sNfL *n* = 16, sGFAP *n* = 15), fatigue (sNfL *n* = 21, sGFAP *n* = 14), anosmia (sNfL *n* = 19, sGFAP *n* = 18) and ageusia (sNfL *n* = 19, sGFAP *n* = 19) prior to a SARS-CoV2 infection and during the Long-COVID period. The Wilcoxon test was used to assess group differences.

	sNfL before Infection vs. after Infection			sGFAP before Infection vs. after Infection		
Symptoms	Median	IQR	*p*-Value	Median	IQR	*p*-Value
headache	6.9 to 6.9	5.8 to 8.4 vs. 5.5 to 7.8	*p* = 0.29	67.7 to 66.9	52.0 to 79.5 vs. 45.2 to 73.5	*p* = 0.71
fatigue	7.3 vs. 6.9	5.8 to 9.3 vs. 5.5 to 8.4	*p* = 0.17	66.8 to 69.9	47.4 to 82.7 vs. 51.8 to 86.0	*p* = 0.39
anosmia	7.3 vs. 6.7	5.3 to 8.8 vs. 50.6 to 8.3	*p* = 0.35	59.0 vs. 60.9	47.9 to 74.6 vs. 50.1 to 70.1	*p* = 0.35
ageusia	6.7 vs. 6.6	5.3 to 8.6 vs. 5.6 to 7.9	*p* = 0.23	61.7 vs. 60.7	49.5 to 76.8 vs. 45.3 to 71.0	*p* = 0.16

**Table 6:** sNfL and sGFAP in the entire cohort in participants with vs. without headache (sNfL *n* = 16 vs. 15, sGFAP *n* = 17 vs. 16), fatigue (sNfL *n* = 21 vs. 12, sGFAP *n* = 22 vs. 11), anosmia (sNfL *n* = 20 vs. 17, sGFAP *n* = 20 vs. 17) and ageusia (sNfL *n* = 20 vs. 14, sGFAP *n* = 20 vs. 15). The Mann–Whitney U Test was used to assess biomarker difference between the groups.

	sNfL			sGFAP		
Symptoms	Median	IQR	*p*-Value	Median	IQR	*p*-Value
headache vs. no headache	6.9 vs. 6.7	5.5 to 7.8 vs. 6.0 to 7.7	*p* = 0.82	68.5 vs. 60.7	49.0 to 83.6 vs. 51.0 to 65.5	*p* = 0.32
fatigue vs. no fatigue	6.9 vs. 6.2	5.5 to 8.4 vs. 5.8 to 6.5	*p* = 0.35	67.7 vs. 60.7	50.1 to 86.0 vs. 53.8 to 66.1	*p* = 0.62
anosmia vs. no anosmia	6.6 vs. 6.6	5.8 to 7.9 vs. 6.0 to 8.0	*p* = 0.56	61.5 vs. 66.8	50.0 to 71.4 vs. 48.5 to 91.2	*p* = 0.53
ageusia vs. no ageusia	6.6 vs. 6.6	5.8 to 7.9 vs. 6.0 to 8.0	*p* = 0.56	61.5 vs. 66.8	50.0 to 71.4 vs. 48.5 to 91.2	*p* = 0.53

## Data Availability

Anonymized data that underlie the results reported in this article are available upon justified request to the corresponding author.
